# Global analysis of protein lysine 2-hydroxyisobutyrylation (K_hib_) profiles in Chinese herb rhubarb (Dahuang)

**DOI:** 10.1186/s12864-021-07847-0

**Published:** 2021-07-15

**Authors:** Tong Qi, Jinping Li, Huifang Wang, Xiaofan Han, Junrong Li, Jinzhe Du

**Affiliations:** 1 Shandong Provincial Key Laboratory of Dryland Farming Technology, College of Agronomy,, Qingdao Agricultural University, Shandong 266109 Qingdao, China; 2 International Cooperation Department of Qilu University of Technology (Shandong Academy of Sciences), Jinan, China; 3 Bathurst Future Agri-Tech Institute of Qingdao Agricultural University, Qingdao, China

**Keywords:** Lysine 2-hydroxyisobutyrylation (K_hib_), Posttranslational modification (PTM), Motif, Chinese herb rhubarb (Dahuang)

## Abstract

**Background:**

Lysine 2-hydroxyisobutyrylation (K_hib_) is a newly discovered protein posttranslational modification (PTM) and is involved in the broad-spectrum regulation of cellular processes that are found in both prokaryotic and eukaryotic cells, including in plants. The Chinese herb rhubarb (Dahuang) is one of the most widely used traditional Chinese medicines in clinical applications. To better understand the physiological activities and mechanism of treating diseases with the herb, it is necessary to conduct intensive research on rhubarb. However, K_hib_ modification has not been reported thus far in rhubarb.

**Results:**

In this study, we performed the first global analysis of K_hib_-modified proteins in rhubarb by using sensitive affinity enrichment combined with high-accuracy HPLC-MS/MS tandem spectrometry. A total of 4333 overlapping K_hib_ modification peptides matched on 1525 K_hib_-containing proteins were identified in three independent tests. Bioinformatics analysis showed that these K_hib_-containing proteins are involved in a wide range of cellular processes, particularly in protein biosynthesis and central carbon metabolism and are distributed mainly in chloroplasts, cytoplasm, nucleus and mitochondria. In addition, the amino acid sequence motif analysis showed that a negatively charged side chain residue (E), a positively charged residue (K), and an uncharged residue with the smallest side chain (G) were strongly preferred around the K_hib_ site, and a total of 13 K_hib_ modification motifs were identified. These identified motifs can be classified into three motif patterns, and some motif patterns are unique to rhubarb and have not been identified in other plants to date.

**Conclusions:**

A total of 4333 K_hib_-modified peptides on 1525 proteins were identified. The K_hib_-modified proteins are mainly distributed in the chloroplast, cytoplasm, nucleus and mitochondria, and involved in a wide range of cellular processes. Moreover, three types of amino acid sequence motif patterns, including EK_hib_/K_hib_E, GK_hib_ and k.kkk….K_hib_….kkkkk, were extracted from a total of 13 K_hib_-modified peptides. This study provides comprehensive K_hib_-proteome resource of rhubarb. The findings from the study contribute to a better understanding of the physiological roles of K_hib_ modification, and the K_hib_ proteome data will facilitate further investigations of the roles and mechanisms of K_hib_ modification in rhubarb.

**Supplementary Information:**

The online version contains supplementary material available at 10.1186/s12864-021-07847-0.

## Introduction

Protein posttranslational modifications (PTMs) are reversible and dynamic modifications of proteins that are crucial to protein maturation, protein structure and function. Currently, PTMs are known to be the most efficient strategies for expanding the functional diversity of proteins and play key roles in diverse biological processes [1, 2]. Among a wide array of types of PTMs, lysine residues of proteins have been found to be the most common modification sites, such as ubiquitination, SUMOylation, methylation, acetylation, butyrylation, succinylation, crotonylation, malonylation, and propionylation at lysine [3]. Recently, lysine 2-hydroxyisobutyrylation (K_hib_), a new type of PTM at lysine, was discovered to be widespread on human and mouse histone proteins, and it is considered to be a new histone mark in eukaryotic cells [4]. Dozens of K_hib_ sites have been identified on histones, and histone K_hib_ has been shown to regulate gene transcription, which is associated with cell differentiationand cell proliferation [4–7].

K_hib_ signals have been detected in HeLa cells, mouse embryonic fibroblasts (MEFs), *Drosophila* S2 cells and yeast *Saccharomyces cerevisiae* cells on both histone and nonhistone proteins [4]. These findings suggest that K_hib_ should be present in nonhistone proteins and should have functions independent of nucleosomes. To date, K_hib_ has been detected in eukaryotes, including human and animal cells [4, 8] and tissues [9], yeast (*S. cerevisiae*) [6], plants (*Physcomitrella patens* [10], *Oryza sativa* [11, 12]), and bacteria, including *Proteus mirabilis* [13–15] and *Toxoplasma gondii* [16]. These findings indicate that K_hib_ exists on histone proteins in the nucleus but also widely exists on nonhistone proteins in various parts of the cells. For example, proteome-wide profiling of K_hib_ identified 6,548 K_hib_ sites on 1,725 substrate proteins in mammalian cells [6, 17].

LC-MS/MS-based qualitative proteome analysis in rice (*O. sativa*) showed that most K_hib_ proteins are located in the cell (38 %), 28 % in organelles, and 15 % in the membrane [12]. Meanwhile, these identified K_hib_ proteins show a variety of functions, with 44 % of the proteins involved in binding, 40 % of proteins involved in catalytic functions, etc. [12]. In summary, based on accumulating proteomic datasets, K_hib_ occurs extensively in proteins and is an evolutionarily conserved PTM observed in various organisms. Therefore, comprehensive detection of K_hib_-modified proteins and modification sites is essential for understanding the physiological functions and regulatory mechanisms of cells.

However, K_hib_ modification has not been reported thus far in the Chinese herb rhubarb. Rhubarb, also named Rhei or Dahuang, belongs to the *Rheum L.* genus from the Polygonaceae family, which is one of the most ancient and important herbs in traditional Chinese medicine and has been widely used in clinical applications for over a thousand years [18, 19]. Currently, rhubarb has been suggested to possess various forms of activity such as antibacterial, anti-inflammatory, antifibrotic, and anticancer activity, and the regulation of gastrointestinal function. Due to its wide range of pharmacological activities, rhubarb has been used in the treatment of several diseases, including severe acute pancreatitis (SAP), sepsis, and chronic renal failure (CRF) [18]. To better understand the physiological activities and mechanism of treating diseases with the herb, it is necessary to conduct intensive research on rhubarb. Studies have suggested that K_hib_ is broadly obtainable in plant cells, and this modification is highly conserved and enriched in various biological processes [11]. Therefore, to investigate the K_hib_ modification in rhubarb and the potential regulatory functions of K_hib_ for its physiological activity, we present the first systematic proteome-wide analysis of K_hib_ modification in rhubarb. Our findings provide a promising platform for the further exploration of K_hib_ modification in physiological processes in rhubarb.

## Materials and methods

### Plant material and cultivation

Plants of rhubarb (*Rheum palmatum* L.) were used in this study. The specimen of the plant has been deposited in Chinese Virtual herbarium with herbarium ID of WUK 520,024. The rhubarb used in our experiments were identified by Zhu Renbin. Rhubarb plants approximately 2 years old were cultivated in nutrient soil for 20 days in a greenhouse at a relative humidity of 50 % and at 22 °C during the day and 18 °C at night. The leaves of the cultivated rhubarb were collected and used for extraction of protein samples.

### Protein extraction, Western blotting and digestion

Whole proteins of rhubarb leaves were extracted by using a modified phenol isolation protocol [20]. In brief, rhubarb tissues were prefrozen in liquid nitrogen and then ground into fine powder with a mortar and pestle in liquid nitrogen and 10 % polyvinylpolypyrrolidone (PVPP). Ground tissues were suspended in 1.6 mL lysis buffer (1 M sucrose, 0.5 M Tris 8.0, 0.1 M KCl, 50 mM ascorbic acid, 1 % NP40, 1 % NaDOC, 10 mM EDTA, 10 mM DTT, and 1 % protease inhibitor cocktail; the final pH was 8.0) and then sonicated on ice for 30 min to promote the dissolution of the sample proteins. The resulting solution was mixed with an equal volume of tris-saturated phenol (pH 8.0) and then homogenized for 10 min. The homogenate was centrifuged for 10 min at 16,000 g, and the upper phenol phase was collected. The collection of phenol was mixed with four volumes of precipitation buffer (methanol with 0.1 M ammonium acetate, precooled at -20 ℃ before use), and protein precipitation was performed at -20 ℃ overnight. The precipitate was collected by centrifugation at 16,000 g for 10 min, and the pellet was rinsed with cold methanol, repeated three times. The final protein pellet was dissolved in 0.4 mL lysis buffer. The protein concentration was determined by a 2D Quant kit (GE Healthcare).

For Western blotting, a total of 30 µg of protein samples was electrophoresed in a 12 % slab SDS-PAGE gel, and K_hib_-modified proteins were detected with rabbit-derived anti-2-hydroxyisobutyryllysine pan antibody (Micrometer Biotech, China) in a standard procedure. For tryptic digestion, proteins were first reduced by using 5 mM DTT and subsequently alkylated by using 30 mM iodoacetamide (IAM) in a standard procedure. The resulting proteins were suspended in 0.1 M and digested overnight with sequencing grade trypsin (Promega) at a trypsin:substrate ratio of 1:50 (w/w). The tryptic peptides were purified by using a Strata X C_18_ SPE column (Phenomenex) and vacuum-dried before affinity enrichment.

### Immunoaffinity enrichment of K_hib_-modified peptides

The immunoaffinity enrichment of K_hib_-modified peptides were performed as described previously [11, 12]. Briefly, the dried peptides were dissolved in NETN buffer (100 mM NaCl, 1 mM EDTA, 50 mM tris-HCl, 0.5 % NP-40, pH 8.0) and incubated with anti-2-hydroxyisobutyryllysine pan antibody conjugated to agarose beads (Micrometer Biotech, China) overnight with gentle oscillation at 4 °C. Then, the agarose beads were washed four times with NETN buffer and once with ice-cold deionized water. K_hib_-modified peptides were eluted by using 0.1 % trifluoroacetic acid (TFA) and desalted with a C18 ZipTips column (EMD Millipore).

### HPLC-MS/MS analysis

HPLC-MS/MS analysis was carried out using a nano HPLC-MS/MS (UltiMate RSLCnano 3000, Thermo Scientific) coupled to a Q Exactive HF-X mass spectrometer (Thermo Scientific). The enriched K_hib_ peptides were dissolved in HPLC buffer A (0.1 % (v/v) formic acid in water) and then separated by a C18 capillary column (Acclaim PepMap RSLC, 75 μm × 50 cm, 2 μm particles, Thermo Fisher Scientific) at a flow rate of 250 nL/min with a 62 min HPLC gradient (2–10 % buffer B (0.1 % formic acid and 80 % acetonitrile in water) in 6 min, 10–20 % buffer B in 45 min, 20–80 % buffer B in 7 min and 80 % buffer B for 4 min). The eluted peptides were then ionized and electrosprayed (2.0 kV voltage) into the mass spectrometer. The mass spectrometric analysis was performed in data-dependent mode with an automatic switch between a full MS scan and an MS/MS scan in the Orbitrap. Peptides were detected in MS at a resolution of 60,000 with a scan range of 350–1800 m/z and with automatic gain control (AGC) of 1e6. The top 15 peaks were selected for MS/MS by higher-energy collision dissociation (HCD) with a normalized collision energy (NCE) of 26 %. The MS/MS spectra were acquired at a resolution of 30,000. The dynamic exclusion for the data-dependent scan was 6 s, and AGC was set at 1E5. HPLC-MS/MS analysis was performed by Micrometer Biotech Company (Hangzhou, China).

### Database search

The resulting MS/MS raw data were searched against the *Rheum palmatum*, L. transcriptome data (NCBI BiopPoject ID: PRJNA714631). The transcriptome data were concatenated using the decoy database using the MaxQuant search engine (v1.5.2.8) with false discovery rates (FDRs) for proteins, peptides and modifications of less than 1 %. Trypsin/P was specified as a cleavage enzyme and allowed a maximum of 4 missed cleavages, 5 modifications per peptide and 5 charges. The mass tolerances for precursor ions and fragment ions were set to 10 ppm and 0.02 Da, respectively. Carbamidomethylation on Cys was specified as a fixed modification, and oxidation on Met, 2-hydroxyisobutyrylation on both Lys and the protein N-terminus were specified as variable modifications. The minimal peptide length was set to 7. K_hib_ sites identified with a localization probability of < 0.75 were removed. The cut-off score for modified peptides was set to 40.

### Bioinformatics analysis of K_hib_-modified peptides and proteins

The bioinformatics analysis of the identified K_hib_**-**modified peptides and proteins was performed as described in detail previously [11]. Briefly, amino acid sequence motifs (10 amino acids upstream or downstream of K_hib_ sites) were analysed by using motif-X software (http://www.motif-x.med.harvard.edu). Motif-based clustering analysis was also performed, and cluster membership was visualized by using a heat map via the “heatmap.2” function in the “gplots” R package.

The Gene Ontology (GO) annotation of disentitled proteins was derived from the UniProt-GOA database (http://www.ebi.ac.uk/GOA/). Proteins were classified into three categories (biological process, cellular compartment, and molecular function) based on GO annotation. The InterPro database was searched for proteins that were not annotated by the UniProt-GOA database. The Kyoto Encyclopedia of Genes and Genomes (KEGG) [21] Automatic Annotation Server (KAAS) tool was used to identify enriched pathways, and the resulting results were mapped to the KEGG pathway database by using the online service tool KEGG mapper that provided by Kanehisa Laboratories (http://www.kegg.jp/kegg/kegg1.html). The functional annotation tool of DAVID bioinformatics resources 6.7 was used for GO and KEGG pathway enrichment analysis. Fisher’s exact test (two-tailed test) was applied to test for GO/KEGG pathway enrichment analysis. Correction for multiple hypothesis testing was performed by using standard FDR control methods, and annotation terms with a corrected p-value < 0.05 were considered significant. Additionally, the subcellular localization of identified proteins was predicted by the WoLF PSORT platform (https://wolfpsort.hgc.jp/). The domain functional descriptions of the identified proteins were annotated using the sequence analysis application InterProScan (http://www.ebi.ac.uk/interpro/) based on the protein sequence alignment method, and the InterPro domain database (a resource that provides functional analysis of protein sequences by classifying them into families and predicting the presence of domains and important sites) was used.

## Results and discussion

### Proteome-wide identification of K_hib_ modification in rhubarb

Lysine K_hib_ modification is a novel protein PTM initially identified on histones and involved in transcriptional activity regulation in mammals [4]. In recent years, this modification has been identified in diverse organisms, including eukaryotes and prokaryotes [4, 10, 14, 22]. However, the K_hib_ modification has not been revealed in rhubarb, a type of widely used traditional Chinese medicine in clinical application in China. In this study, to detect the K_hib_ modification status in rhubarb, total protein samples were isolated from rhubarb leaves and submitted to proteome-wide identification conducted by combining affinity enrichment by the K_hib_ antibody with nano-HPLC-MS/MS.

**Fig. 1:**
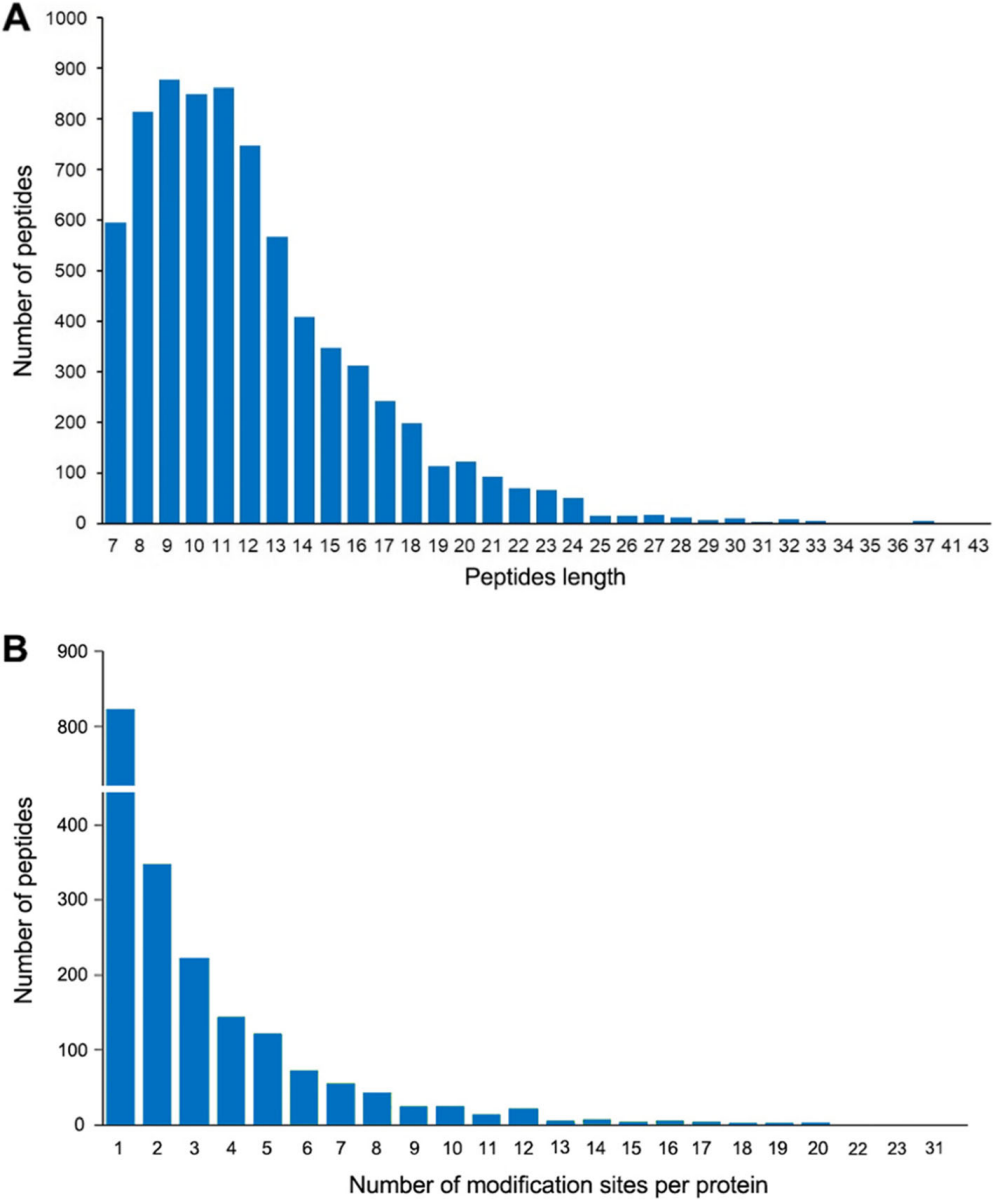
Distribution of identified lysine 2-hydroxyisobutyrylation (K_hib_) peptides and proteins. (**A**) Distribution of K_hib_-modified peptides based on their length; (**B**) Distribution of K_hib_-modified proteins based on the number of K_hib_ peptides identified

To obtain more accurate data, we performed three independent tests, and 6062, 7594 and 7660 K_hib_-modified peptides were separately identified with peptide score > 40, localization probability > 0.75 and most peptide mass error < 2 ppm (the detailed identification information is shown in Supplemental Table S1 and Fig. S1). Overall, a total of 4333 peptides that overlapped in the three tests were identified to be K_hib_-modified. The lengths of the identified peptides varied from 7 to 43 amide acid residues, but most peptide lengths were between 7 and 20 residues (Fig. 1 A). These K_hib_-modified peptides matched 1525 proteins with K_hib_ sites from 1 to 31 (Fig. 1 B). Although most proteins contain only one K_hib_ site, 4 proteins were found to have more than 20 K_hib_ sites, including elongation factor 2-like (22 sites), hypothetical protein CCACVL1_04477 (22 sites), ribosomal protein (23 sites), and heat shock cognate protein 80 (Supplemental Table S1), indicating that K_hib_ is a type of multiple PTM. The distribution of modified peptide lengths and modified sites within individual proteins was similar to previous reports [23, 24]. Based on the results described above, K_hib_ modification is an abundant and complex PTM and may have a very important regulatory effect on protein function in plants, which is consistent with previous reports of the K_hib_ modification proteome identified in rice [11].

### Characterization of K_hib_ modification proteins identified in rhubarb

**Fig. 2:**
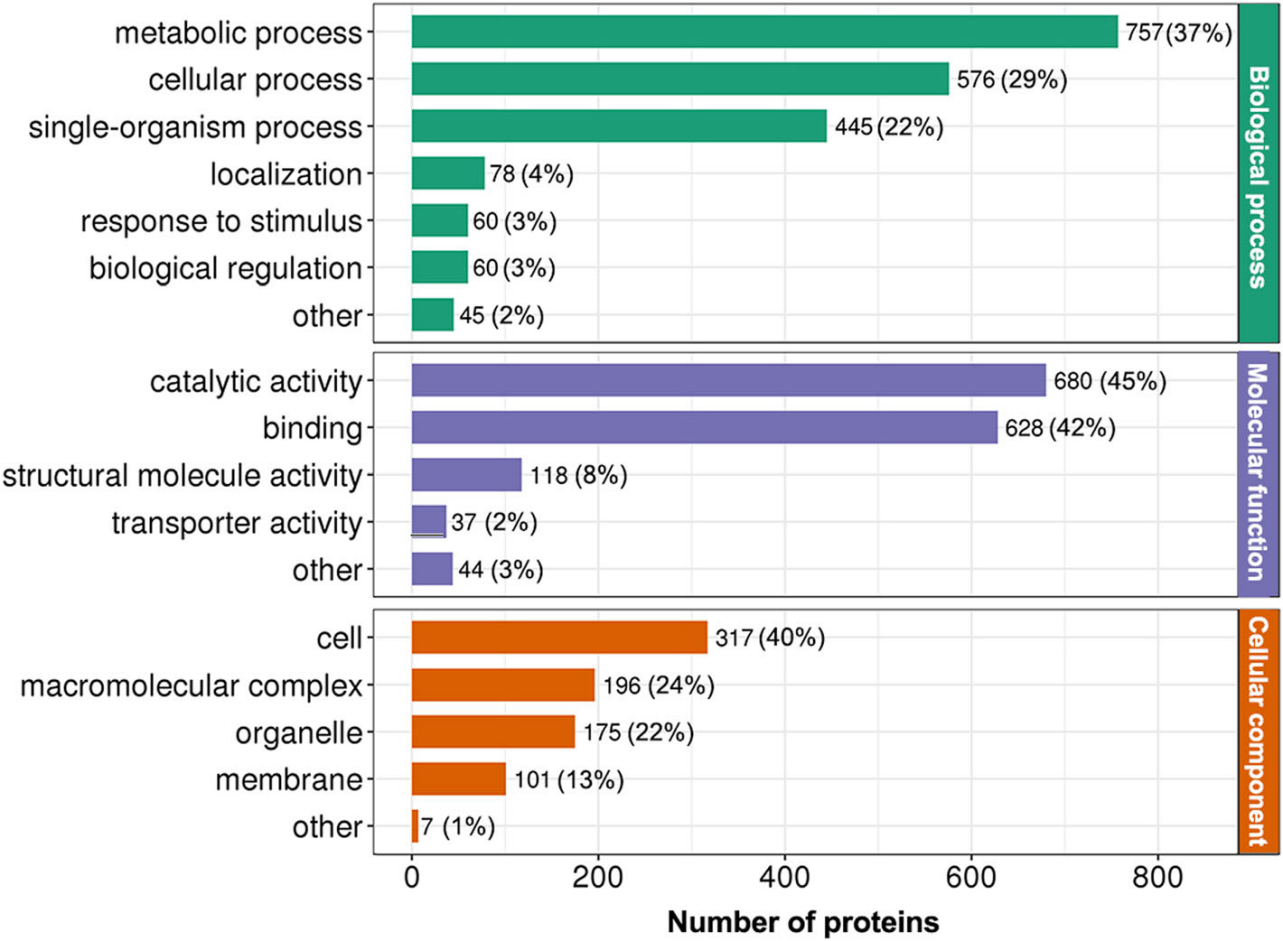
GO functional classification of the K_hib_-modified proteins based on biological process, molecular function, and cellular component. The number of proteins and the percentages of each category are indicated

Gene ontology (GO) classifications are often used to help analyse the potential functions of proteins [25]. Therefore, to better understand the potential roles of K_hib_-modification in rhubarb, all identified K_hib_-modified proteins were submitted to GO functional classification analysis based on their biological process, cellular component, and molecular function. As shown in Fig. 2 and Supplemental Table S2, the classification results showed that the K_hib_-modified proteins were involved in a variety of biological processes, cellular components and molecular functions. In the classification of the GO term “biological process”, two major classes of K_hib_-modified proteins were classified into metabolic process and cellular process, accounting for 37 and 29 % of the total K_hib_-modified proteins, respectively (Fig. 2, indicated in green). In the classification of the GO term “molecular function”, most of the K_hib_-modified proteins were classified into two categories, catalytic activity (45 %) and binding (44 %) (Fig. 2, indicated in purple). This result was in good agreement with the classification result of the biological process, and these two results suggest that the enzymatic proteins associated with metabolism and regulatory proteins related to cellular processes are more likely to be K_hib_-modified in rhubarb. In the “cellular components” category of GO classification, 40 % of all identified K_hib_ proteins were in the cell, 24 % in macromolecular complex, 22 % in the organelle and 13 % in the membrane (Fig. 2, indicated in orange).

**Fig. 3:**
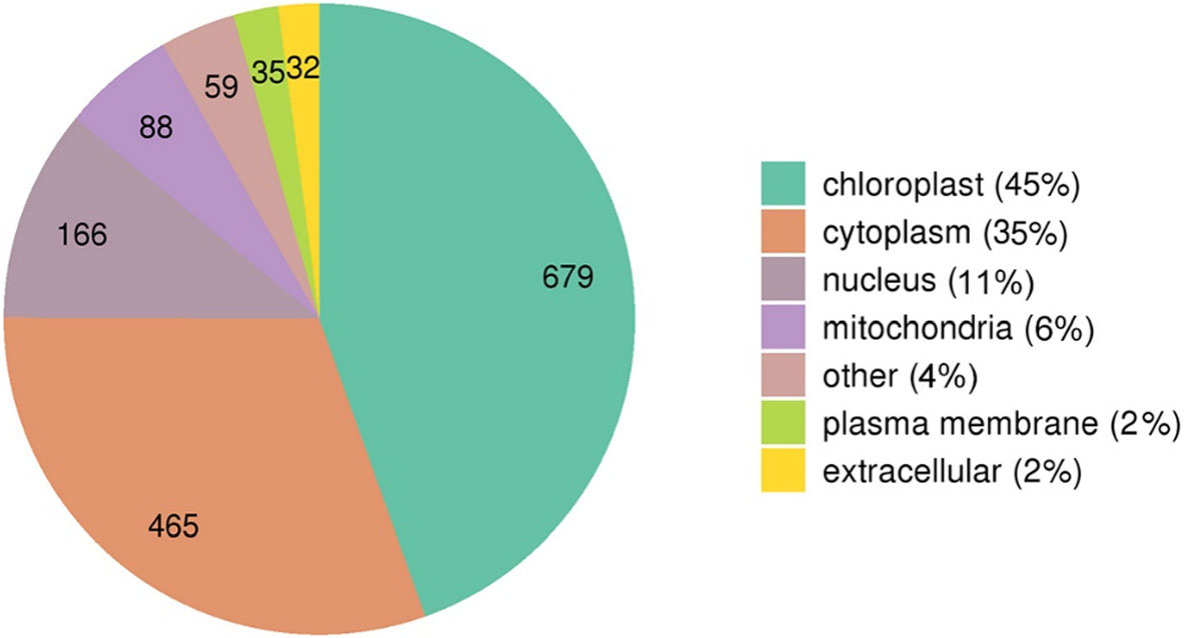
Subcellular localization distribution of K_hib_-modified proteins

The subcellular localizations of K_hib_-modified proteins were also analysed, and the results showed that most of the proteins were distributed in the chloroplast (45 %), cytoplasm (35 %), nucleus (11 %) and mitochondria (6 %) (Fig. 3 and Supplemental Table S3).

The overall trend of GO classification (including biological process, molecular function and cellular components) and subcellular localization of K_hib_ proteins identified in rhubarb were similar to previous findings obtained in other plants such as *P. patens* [10] and rice [12], although the percentages of each category were slightly different.

### GO, KEGG Pathway and Domain Enrichment Analysis of K_hib_-modified Proteins

**Fig. 4:**
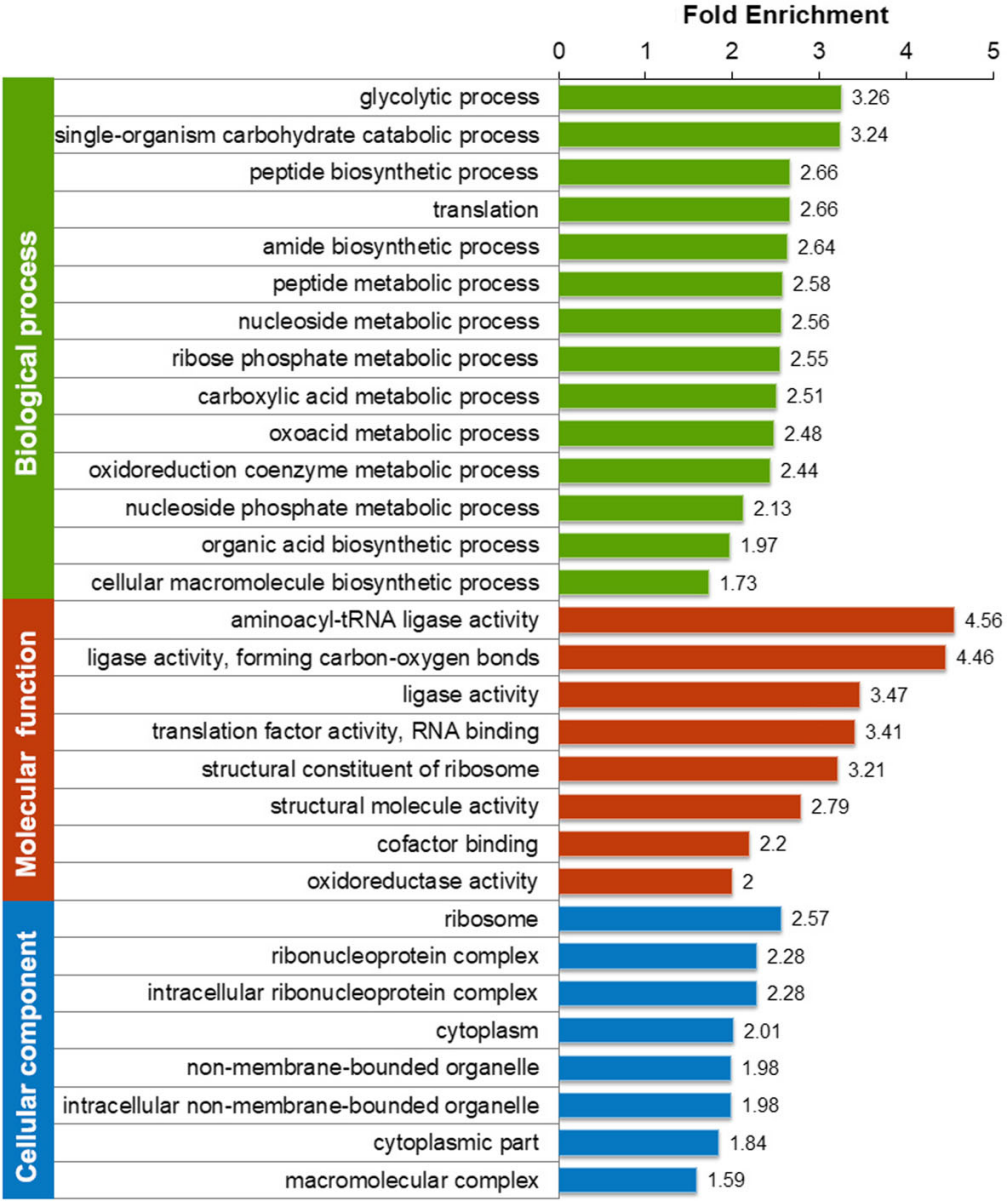
GO enrichment analysis of the K_hib_-modified proteins. The y-axis denotes the categories of GO terms. The x-axis denotes the fold enrichment

To investigate which categories of proteins are preferred targets for K_hib_ modification, the GO, KEGG pathway and protein domain enrichments of the K_hib_ modification proteome obtained in rhubarb were analysed, and the significantly enriched categories for biological process, molecular function and cellular component of K_hib_-modified proteins are shown in Fig. 4 and Supplemental Table S4. In the GO enrichment of biological process, the terms glycolytic process, single-organism carbohydrate catabolic process, peptide biosynthetic process, translation, amide biosynthetic process and peptide metabolic process were found to be significantly enriched (Fig. 4, indicated in green bars). The enrichment of these biological processes suggests that those proteins associated with carbohydrate metabolism and protein biosynthesis may be preferred to be K_hib_-modified in rhubarb. Similar observations were also found in the GO enrichment analysis for the molecular function, in which proteins possessing aminoacyl-tRNA ligase activity, ligase activity, translation factor activity and structural constituent of ribosome were highly enriched (Fig. 4, indicated in red bars). These enriched proteins are mainly related to protein biosynthesis. Regarding the GO enrichment analysis for the cellular component, the K_hib_-modified proteins were significantly enriched in ribosomes, ribonucleoprotein complexes and intracellular ribonucleoprotein complexes (Fig. 4, indicated in blue bars). Overall, the results of GO enrichment analysis strongly suggest that those proteins related to protein biosynthesis and central metabolism may be strictly regulated by K_hib_ modification.

**Fig. 5:**
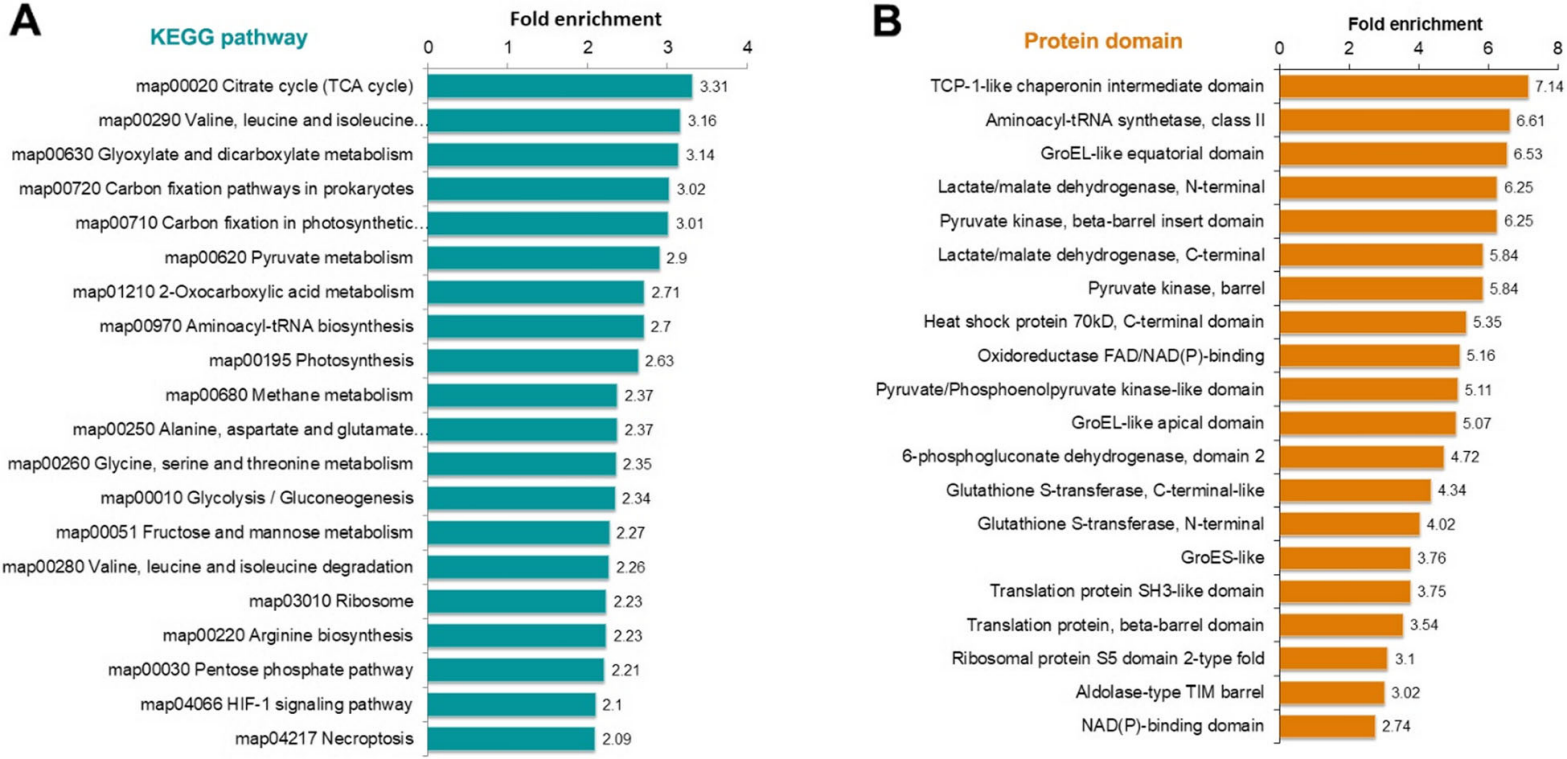
KEGG pathway and protein domain enrichment analysis of the K_hib_-modified proteins. The y-axis denotes the categories of KEGG pathways (A) and protein domains (B). The x-axis denotes the fold enrichment.

**Fig. 6:**
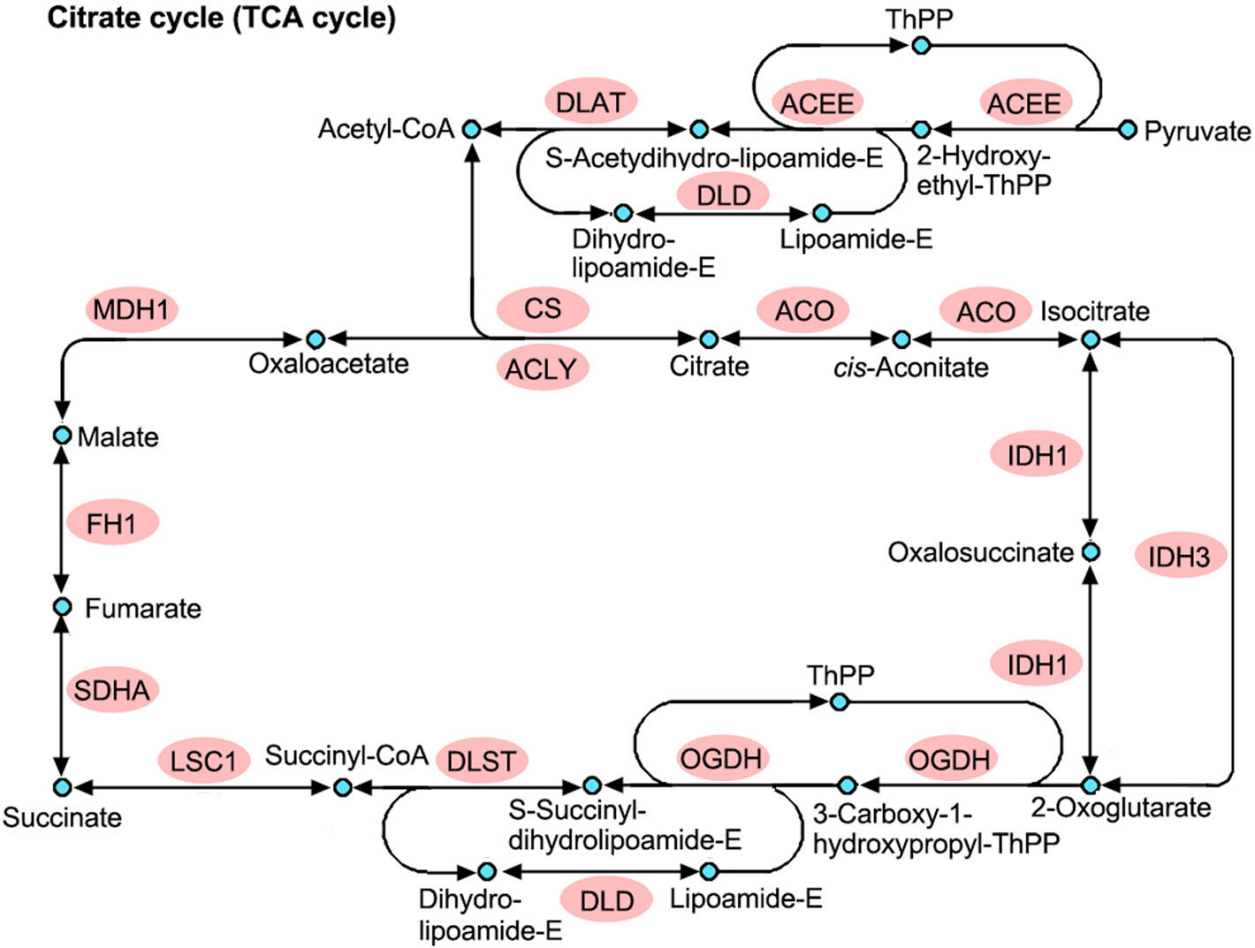
K_hib_-modified enzymes enriched in the TCA cycle in rhubarb. The identified K_hib_ enzymes are indicated with a red background

Consistent with these findings, the enrichment analysis of KEGG pathway and protein domains also showed that proteins related to protein synthesis and central metabolism tended to be K_hib_-modified (Fig. 5, Supplemental Table S5 and Table S6). As shown in Fig. 5 A and Supplemental Table S5, enrichment analysis of KEGG pathways demonstrated that K_hib_-modified proteins were enriched in the pathways of protein synthesis, including valine, leucine and isoleucine biosynthesis, aminoacyl-tRNA biosynthesis, and ribosome (K_hib_-modified proteins associated with the ribosome are shown in a schematic diagram, as shown in Supplemental Fig. S5). In addition to the enrichment of K_hib_ proteins associated with protein biosynthesis, proteins related to central metabolic processes, including the citrate cycle (TCA cycle), glycolysis/gluconeogenesis, pentose phosphate pathway, photosynthesis, carbon fixation in photosynthetic organisms, glyoxylate and dicarboxylate metabolism, and pyruvate metabolism, were also found to be highly enriched (Fig. 5 A and Supplemental Table S5). Most of the enzymatic proteins involved in glycolysis/gluconeogenesis, the pentose phosphate pathway, the TCA cycle and oxidative phosphorylation were identified to be K_hib_-modified (Fig. 6 shows the K_hib_-modified enzymes in the TCA cycle, and Supplemental Fig. S6 shows the modified enzymes in glycolysis/gluconeogenesis, the pentose phosphate pathway and oxidative phosphorylation).

The results of protein domain enrichment analysis showed that proteins possessing the domains of TCP-1-like chaperonin intermediate domain, aminoacyl-tRNA synthetase (class II), GroEL-like equatorial domain, lactate/malate dehydrogenase (both N-terminal and C-terminal), pyruvate kinase (beta-barrel insert domain), pyruvate kinase (barrel), etc., were found to be highly enriched (Fig. 5 B and Supplemental Table S6). It is not difficult to find that these enriched protein domains are involved in two main physiological functions: protein biosynthesis and central metabolism. In combination, the enrichment analysis of GO annotation, KEGG pathway and protein domain demonstrates that proteins with K_hib_ modification in relation to protein biosynthesis and central metabolism are extremely important for the physiological activities of rhubarb.

Notably, the finding that the K_hib_ modification proteins mainly involved in central metabolism and protein biosynthesis are significantly enriched has also been identified in rice [11, 12]. In addition to the K_hib_ modification in plants, other types of PTMs on lysine in other organisms have also been found to tend to occur on proteins closely related to protein biosynthesis and central metabolism such as lysine acetylation and succinylation in *Vibrio parahaemolyticus* [3, 26] and *Mycobacterium tuberculosis* [27, 28] and lysine malonylation in *Escherichia coli* [29]. These findings indicate the fundamental role of lysine modification in different organisms, including eukaryotes and prokaryotes.

### Analysis of K_hib_-modified peptides reveals conserved motifs

**Fig. 7:**
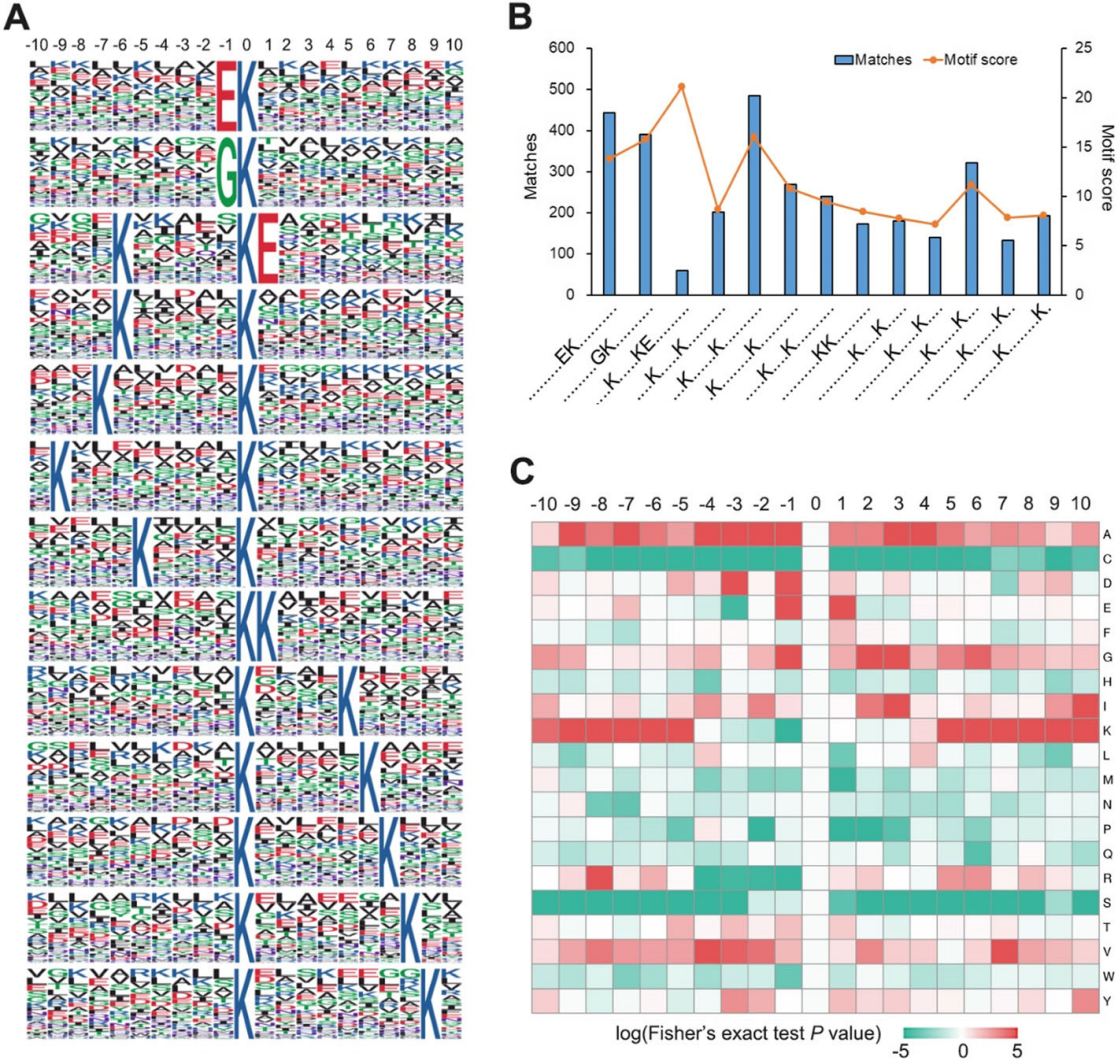
Properties of the identified K_hib_ peptides. (**A**) The K_hib_ sequence motifs and conservation of K_hib_ sites. The size of the letter indicates the frequency of the amino acid residing at that position. (**B**) Frequency of each motif occurring in peptides with K_hib_ modification. Motif score was also indicated in the histogram. (**C**) Heat map showing the frequency of the amino acid residues around the K_hib_ sites. The enrichment [11] and dispersion (green) of amino acids from −10 to +10 flanking the K_hib_ sites.

Previous studies on both eukaryotic and prokaryotic cells have found a preference for specific amino acid residues around the K_hib_ site [10–12, 14]. Therefore, we utilized the Motif-X tool, software for extracting overrepresented patterns [30], to further analyse the conserved sequence motifs from all of the K_hib_-modified peptides in rhubarb. Figure 7 and Supplemental Table S7 show that a total of 13 conserved motifs, with amino acid residues from − 10 to + 10 surrounding the K_hib_ site, were extracted from 3234 K_hib_-modified peptides. Survey of these motifs showed that only three amino acid residues are preferred flanking the K_hib_ site: a negatively charged side chain residue of glutamic acid (E), a positively charged residue of lysine (K), and an uncharged residue with the smallest side chain of glycine (G). According to the amino acid residues and their position around the K_hib_ site, these 13 motifs may be classified into three patterns: the + 1 or -1 position is a negatively charged side chain residue (EK_hib_ or K_hib_E), the − 1 position is an uncharged residue with the smallest side chain (GK_hib_), the position of + 5 to + 9 or -5 to -9 (except − 8) is a positively charged residue (k.kkk….K_hib_….kkkkk) (“.” indicates a random amino acid residue, and “k” indicates that a residue at this position in the third pattern of motifs is K). Although a similar motif of EK_hib_ has also been identified in rice [11], the other two motif patterns are unique and are not currently identified in other plants, such as *P. patens* and *O. sativa* (Supplemental Fig. S7). Thus, the preference for K and G flanking K_hib_-modified sites should be a unique feature of catalytic enzymes for K_hib_ modification in rhubarb.

Furthermore, based on the frequency analysis of amino acid residues flanking the K_hib_-modified site, as shown in the heat map (Fig. 7 C), aspartic acid (D) at -3, E at + 1 or -1, G at + 2 or + 3, isoleucine (I) at + 3 or + 10, arginine (R) at -8, valine (V) at -4 or + 6, and alanine (A) at several positions were overpresented around the K_hib_ site. However, cysteine (C), histidine (H), methionine (M) asparagine (N), arginine (Q) serine (S), proline (P) and tryptophan (W) were all underpresented flanking the K_hib_ site. Overall, motif analysis provides a better method to understand potential catalytic enzymes in cells.

## Conclusions

In this study, we presented a proteome-wide identification of K_hib_ modification in rhubarb, an important model of traditional Chinese medical plants, by using a highly sensitive proteomic method. A total of 4333 overlapping K_hib_-modified peptides on 1525 proteins were identified in three independent tests. The K_hib_-modified proteins were evaluated to be distributed mainly in chloroplasts, cytoplasm, nucleus and mitochondria and involved in a wide range of cellular processes through extensive characterization of the K_hib_ proteome. However, the K_hib_-modified proteins in relation to protein biosynthesis and central metabolism were highly enriched, suggesting that they play particularly important physiological roles in rhubarb. Moreover, the analysis of amino acid sequence motifs revealed that glutamic acid (E), glycine (G), and lysine (K) have a strong bias around the K_hib_ site, and three types of motif patterns were extracted from a total of 13 K_hib_-modified peptides. Importantly, some motif patterns unique to rhubarb have been identified, and these motif patterns have not been identified in other plants to date. Although the regulatory effects and mechanisms of K_hib_ modification remain to be clarified, our systematic analysis of K_hib_ modification provides a better understanding of the potential physiological activities of K_hib_-modified proteins in rhubarb.

## Supplementary Information

**Figure S1**, Mass accuracy of MS; **Figure S2**, The bubble chart shows the fold enrichment for GO analyses of K_hib_-modified proteins in terms of biological process, cellular component and molecular function; **Figure S3**, The bubble chart shows the fold enrichment of KEGG pathways of K_hib_-modified proteins; **Figure S4**, The bubble chart shows the fold enrichment of the protein domains of K_hib_-modified proteins; **Figure S5**, Pathway map of the K_hib_-modified enzymes in central carbon metabolism in rhubarb. Central carbon metabolism includes glycolysis/gluconeogenesis, the pentose phosphate pathway, the TCA cycle and oxidative phosphorylation. The identified K_hib_-modified enzymes are indicated in red boxes. The K_hib_-modified enzymes in the TCA cycle are shown in Fig. 6; **Figure S6**, Schematic diagram showing the association of K_hib_-modified proteins with the ribosome in rhubarb. The identified K_hib_-modified proteins are indicated in red boxes.

**Table S1**, All identified K_hib_-modified peptides and proteins in rhubarb.

**Table S2**, GO functional classification of K_hib_-modified proteins.

**Table S3**, Classification of subcellular localizations of K_hib_-modified proteins.

**Table S4**, GO enrichment analysis of K_hib_-modified proteins.

**Table S5**, KEGG pathway enrichment analysis of K_hib_-modified proteins.

**Table S6**, Protein domain enrichment analysis of K_hib_-modified proteins

**Table S7**, Motif analysis of K_hib_ modified peptides.

## Data Availability

The mass spectrometry proteomics data were deposited to the ProteomeXchange Consortium via the PRIDE partner repository with the dataset identifier of PXD026307 (http://www.ebi.ac.uk/pride/archive/projects/PXD026307). The transcriptome data were deposited in n the National Center for Biotechnology Information (NCBI) database (https://www.ncbi.nlm.nih.gov/bioproject/PRJNA714631) (BioProject ID: PRJNA714631).

## Abbreviations

IDH1: Isocitrate dehydrogenase
IDH3: Isocitrate dehydrogenase (NAD^+^)
ACO: Aconitate hydratase
DLAT: Pyruvate dehydrogenase E2 component (dihydrolipoamide acetyltransferase)
ACEE: Pyruvate dehydrogenase E1 component
DLD: Dihydrolipoamide dehydrogenase
CS: Citrate synthase
ACLY: ATP citrate (pro-S)-lyase
MDH1: Malate dehydrogenase
FH1: Fumarate hydratase, class I
SDHA: Succinate dehydrogenase (ubiquinone) flavoprotein subunit
LSC1: Succinyl-CoA synthetase alpha subunit
DLST: 2-oxoglutarate dehydrogenase E2 component (dihydrolipoamide succinyltransferase)
OGDH: 2-oxoglutarate dehydrogenase E1 component
