# Supplementary material for: Global analysis of protein lysine 2-hydroxyisobutyrylation (Khib) profiles in Chinese herb rhubarb (Dahuang)
Source: BMC Genomics. 2021 Jul 15;22:542. doi: 10.1186/s12864-021-07847-0 (PMC8283887; doi:10.1186/s12864-021-07847-0)
Supplement: Supplementary file 1 — Figure S1, Mass accuracy of MS; Figure S2, The bubble chart shows the fold enrichment for GO analyses of Khib-modified proteins in terms of biological process, cellular component and molecular function; Figure S3, The bubble chart shows the fold enrichment of KEGG pathways of Khib-modified proteins; Figure S4, The bubble chart shows the fold enrichment of the protein domains of Khib-modified proteins; Figure S5, Pathway map of the Khib-modified enzymes in central carbon metabolism in rhubarb. Central carbon metabolism includes glycolysis/gluconeogenesis, the pentose phosphate pathway, the TCA cycle and oxidative phosphorylation. The identified Khib-modified enzymes are indicated in red boxes. The Khib-modified enzymes in the TCA cycle are shown in Fig. 6; Figure S6, Schematic diagram showing the association of Khib-modified proteins with the ribosome in rhubarb. The identified Khib-modified proteins are indicated in red boxes. [file 12864_2021_7847_MOESM1_ESM.docx]

**Supplementary information**

**Global Analysis of Protein Lysine 2-Hydroxyisobutyrylation (K_hib_) Profiles in Chinese Herb Rhubarb (Dahuang)**

Tong Qi ^#, 1^, Jinping Li ^#, 2^, Huifang Wang ^1^, Xiaofan Han ^1^, Junrong Li ^3^, Jinzhe Du*^, 1^

*^1^ Shandong Provincial Key Laboratory of Dryland Farming Technology/College of Agronomy, Qingdao AgriculturalUniversity, Qingdao Shandong 266109, China*

*^2^ International Cooperation Department of Qilu University of Technology (Shandong Academy of Sciences)*

*^3^ Bathurst Future Agri-Tech Institute of Qingdao Agricultural University*

***Corresponding author:**

Jinzhe Du, Shandong Provincial Key Laboratory of Dryland Farming Technology/College of Agronomy, Qingdao Agricultural University, Qingdao Shandong 266109, China. wangxx0532@163.com

^#^ These authors contribute equally to this work.

**Supplemental Tables**

**Table S1**. All identified K_hib_-modified peptides and proteins in rhubarb.

**Table S2**. GO functional classification of K_hib_-modified proteins.

**Table S3**. Classification of subcellular localizations of K_hib_-modified proteins.

**Table S4**. GO enrichment analysis of K_hib_-modified proteins.

**Table S5**. KEGG pathway enrichment analysis of K_hib_-modified proteins.

**Table S6**. Protein domain enrichment analysis of K_hib_-modified proteins.

**Table S7.** Motif analysis of K_hib_ modified peptides.

(Note: all supplemental tables are showed in Excel files (.xlsx))

**Supplemental Figure Legends**

**Fig. S1.** Mass accuracy of MS.

**Fig. S2.** The bubble chart shows the fold enrichment for GO analyses of K_hib_-modified proteins in terms of biological process, cellular component and molecular function.

**Fig. S3.** The bubble chart shows the fold enrichment of KEGG pathways of K_hib_-modified proteins.

**Fig. S4.** The bubble chart shows the fold enrichment of the protein domains of K_hib_-modified proteins.

**Fig. S5.** Pathway map of the K_hib_-modified enzymes in central carbon metabolism in rhubarb. Central carbon metabolism includes glycolysis/gluconeogenesis, the pentose phosphate pathway, the TCA cycle and oxidative phosphorylation. The identified K_hib_-modified enzymes are indicated in red boxes. The K_hib_-modified enzymes in the TCA cycle are shown in Figure 6.

**Fig. S6.** Schematic diagram showing the association of K_hib_-modified proteins with the ribosome in rhubarb. The identified K_hib_-modified proteins are indicated in red boxes.

**Fig. S7.** Sequence pattern surrounding the K_hib_ sites that include the significantly enriched and depleted residues based on K_hib_ peptides and non-modification peptides from *P. patens*, *O. sativa* and *R. palmatum* (The K_hib_ sequence pattern of *P. patens* and *O. sativa* were from [7]).

**Supplemental Figures**

**
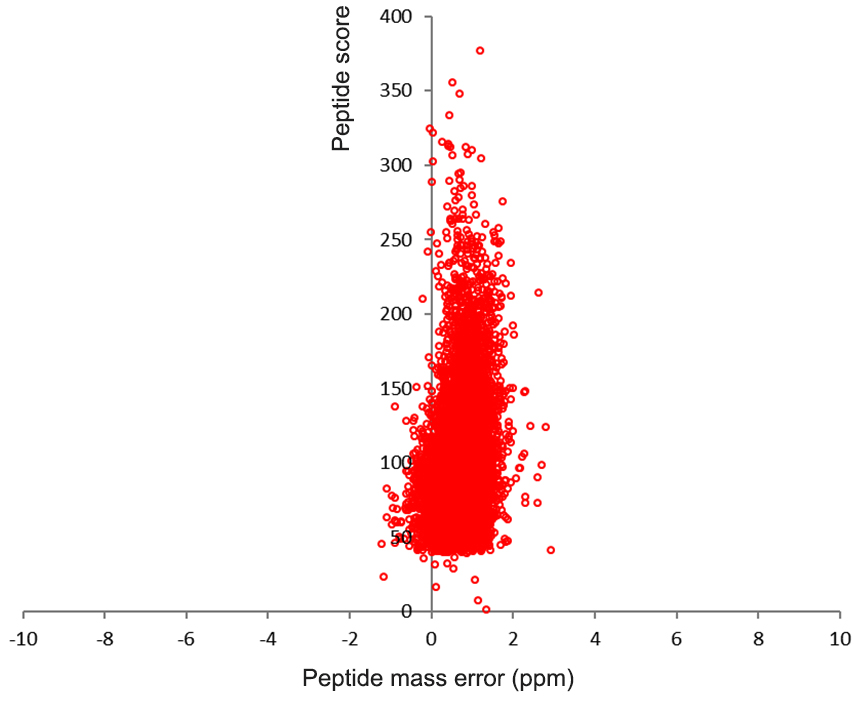
**

**Fig. S1**


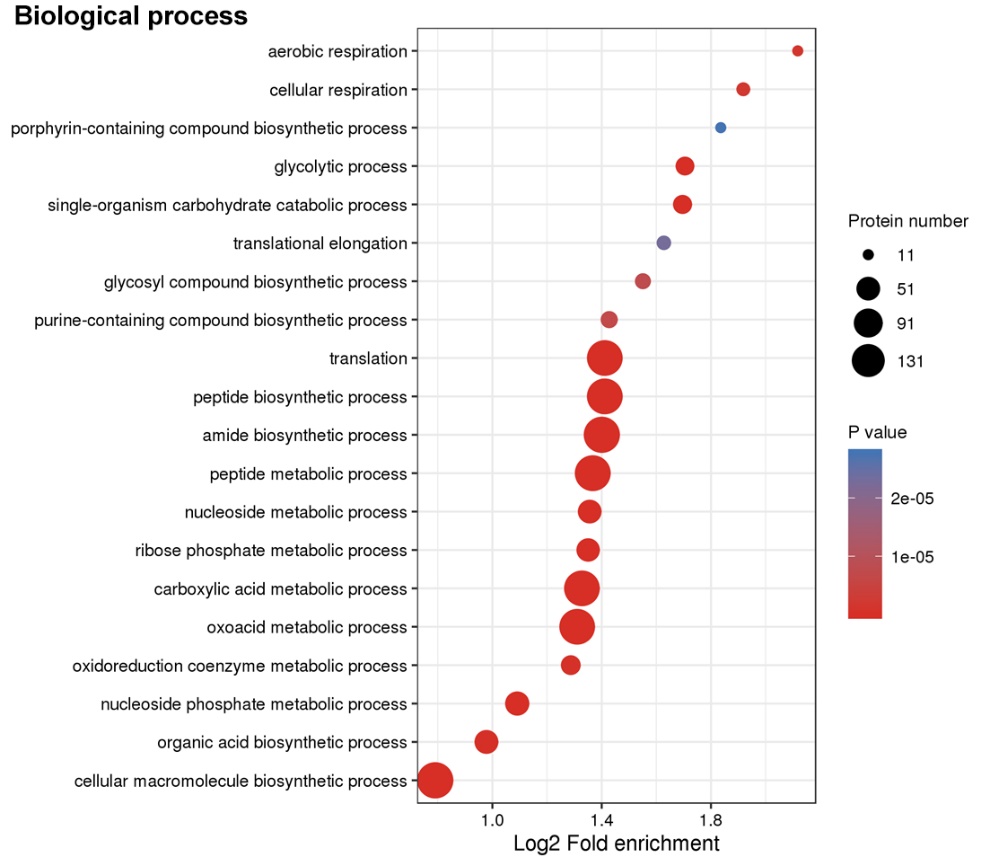


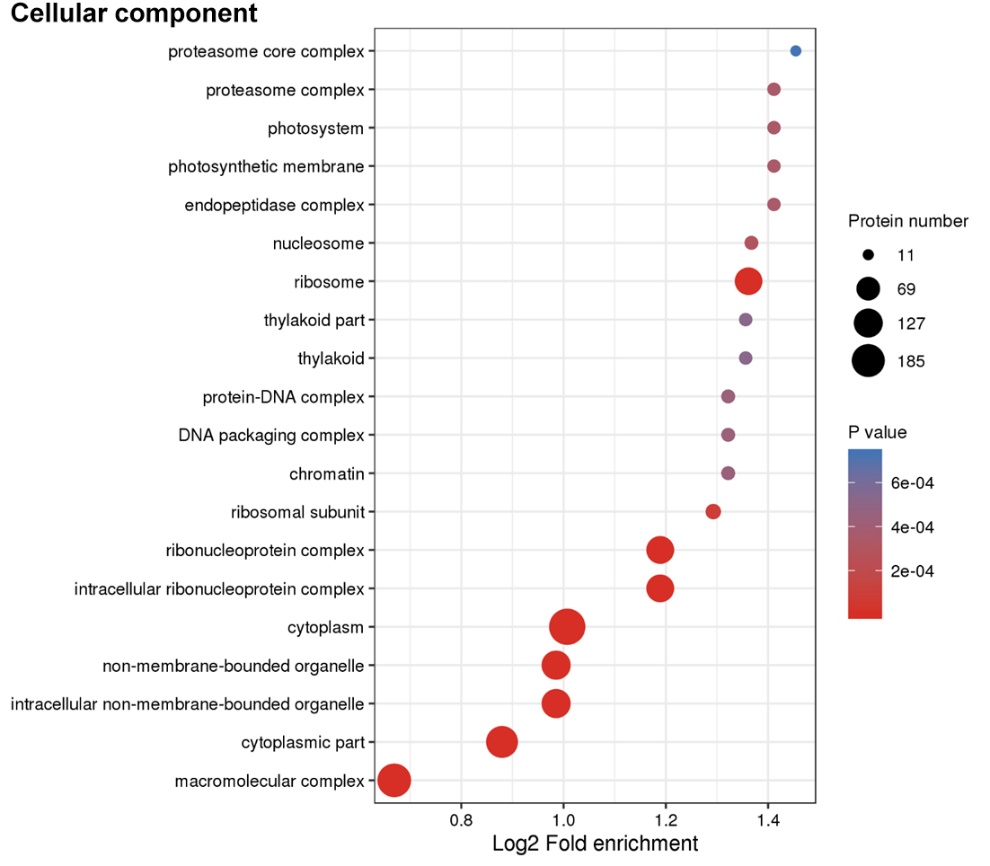


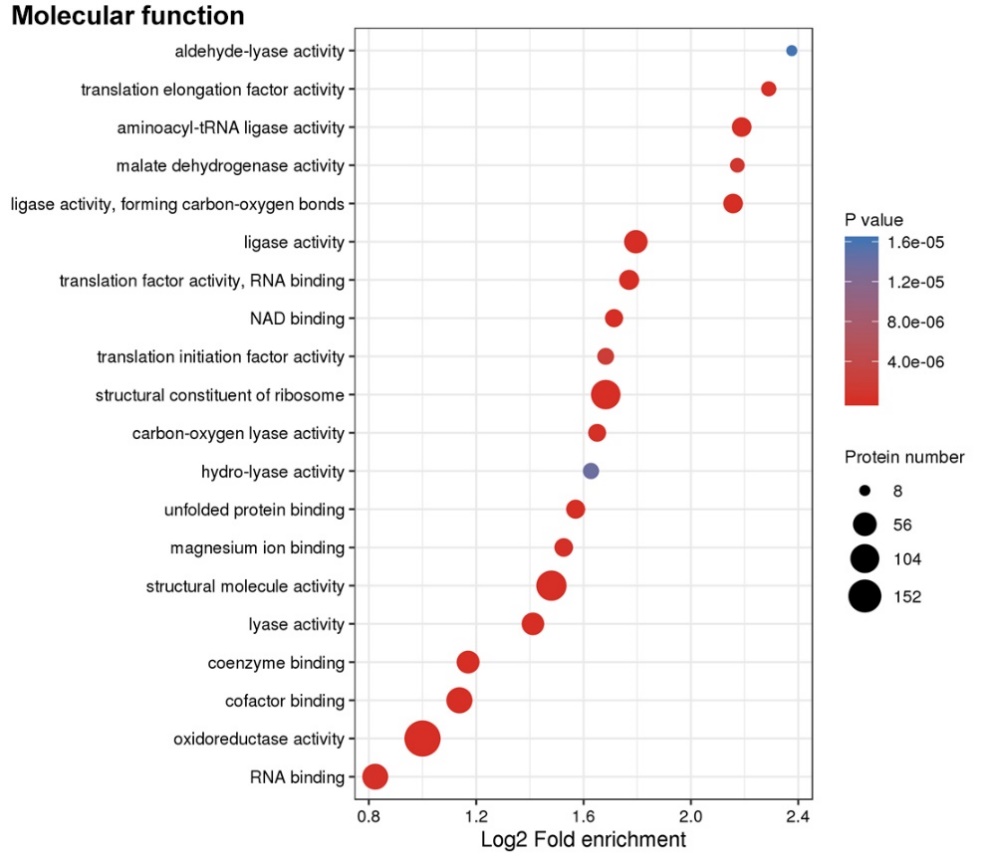


**Fig. S2**


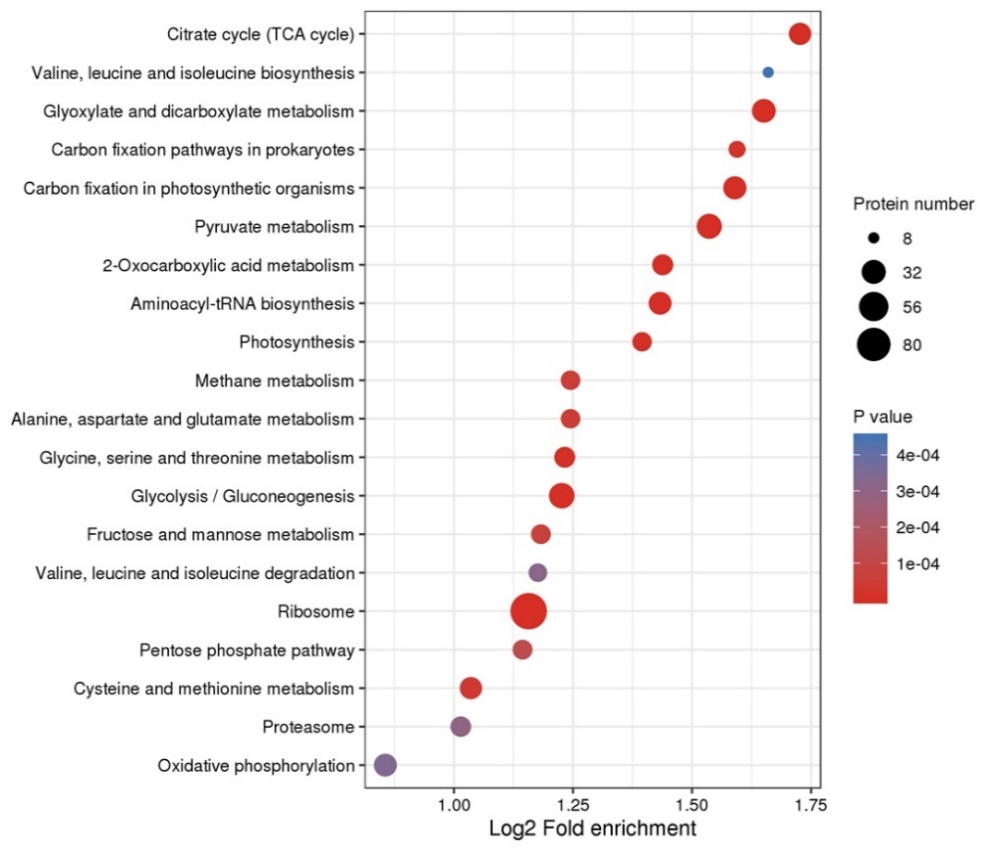


**Fig. S3**


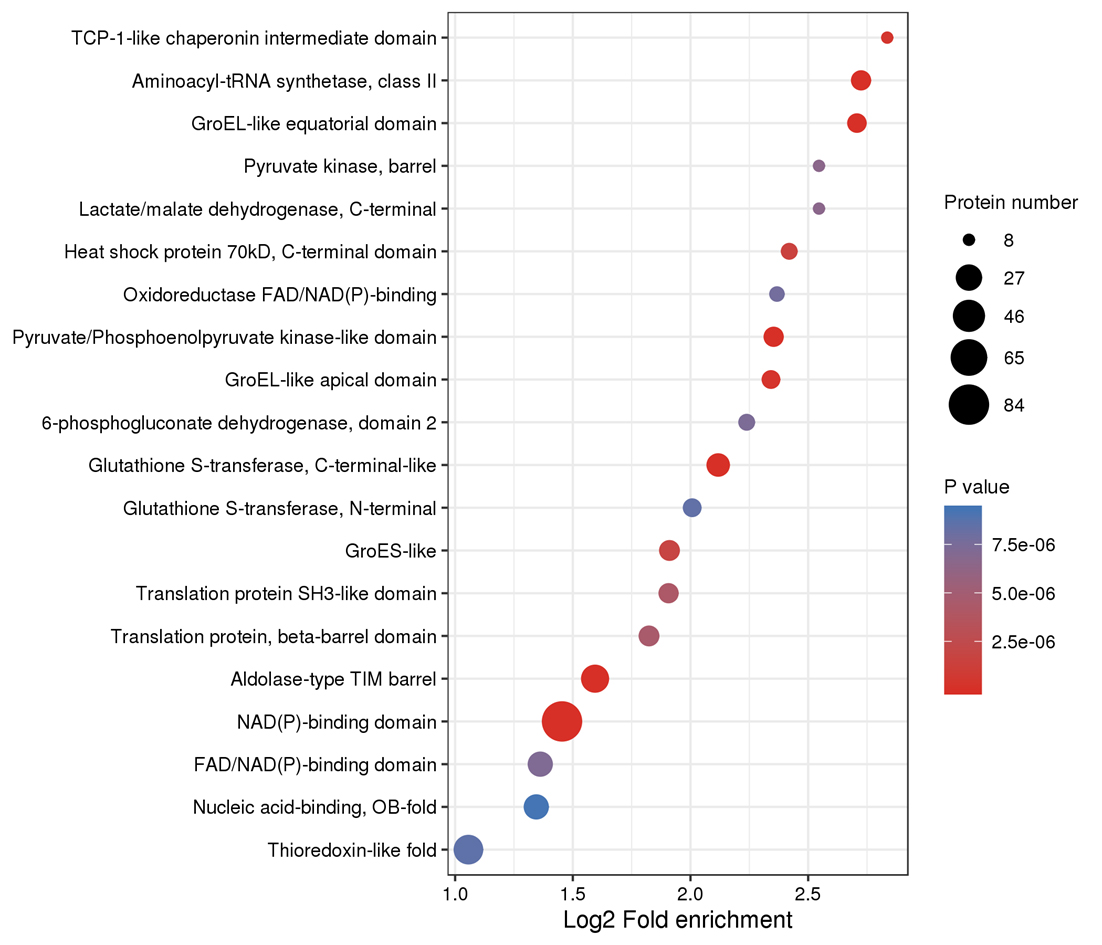


**Fig. S4**


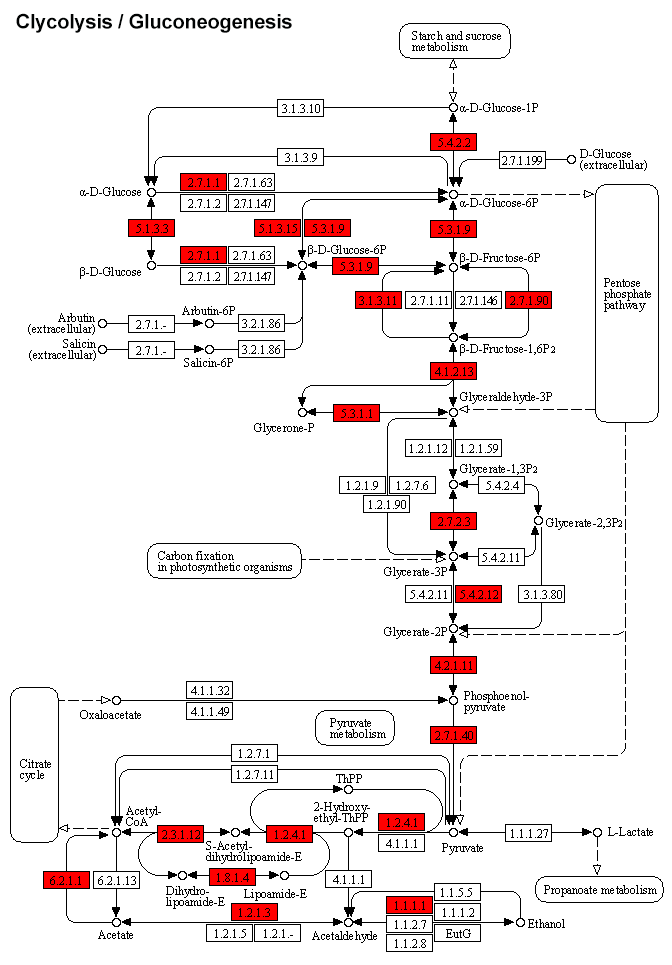


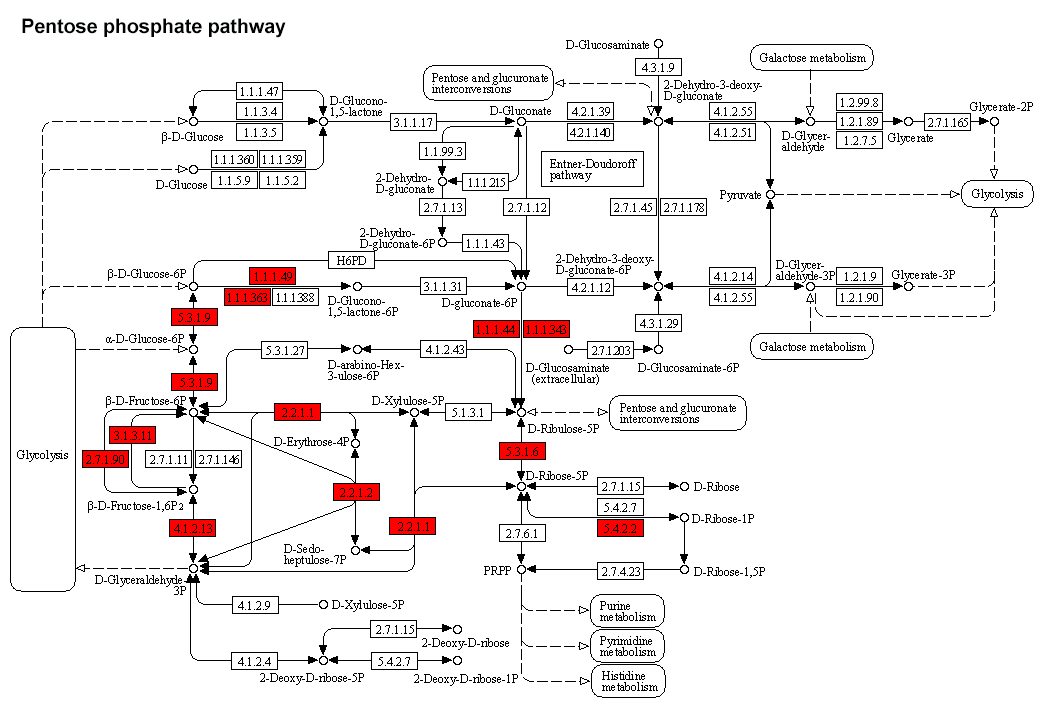


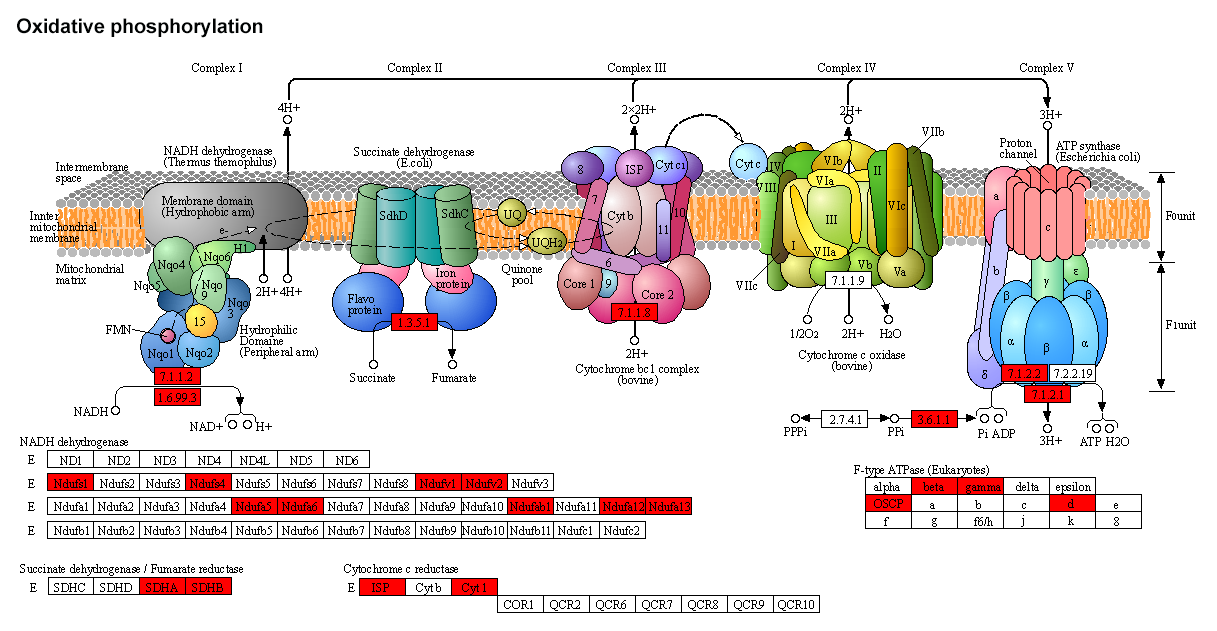


**Fig. S5**


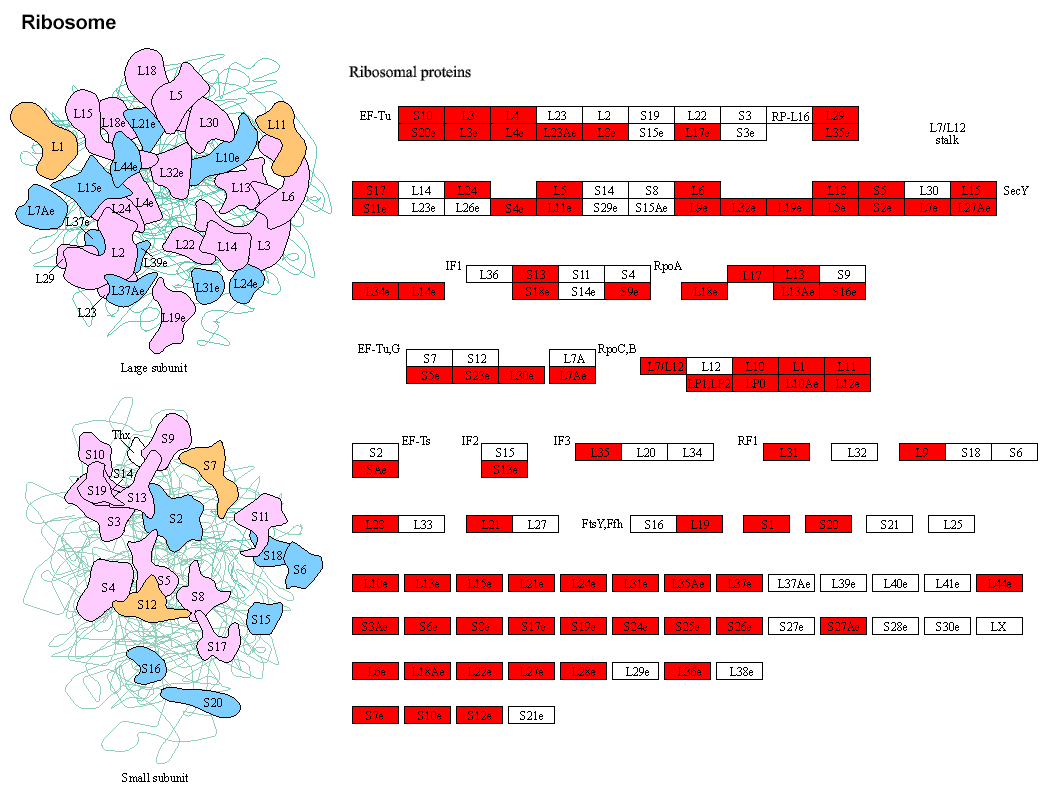


**Fig. S6**

**
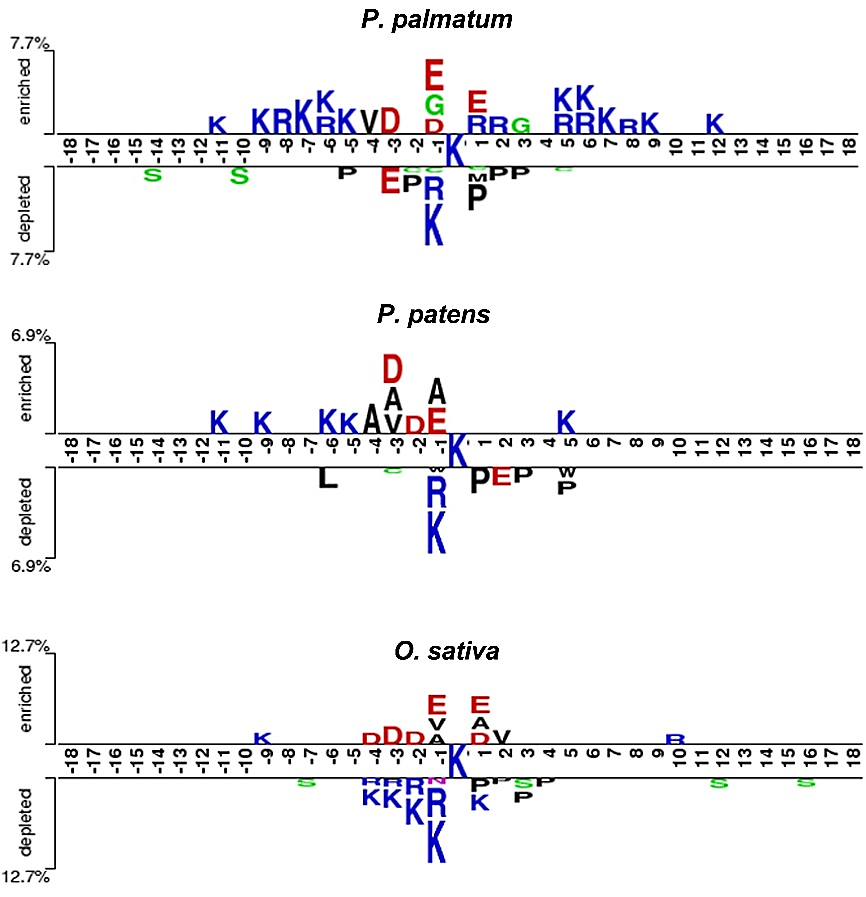
**

**Fig. S7**
